# Comparative evaluation of module-based strategies for controlling lepidopterous and dipterous pests in rice (*Oryza sativa* L.) cultivation

**DOI:** 10.1186/s12870-026-09601-8

**Published:** 2026-08-06

**Authors:** Akanksha N. Humane, D. B. Undirwade, B. N. Chaudhari, U. S. Kulakrni, S. L. Borkar, G. R. Shamkuwar, M. P. Mohril, Sandeep Gawdiya, Ali Salem, Mohamed A. Mattar

**Affiliations:** 1 Department of Agricultural Entomology, Dr. Panjabrao Deshmukh Krishi Vidyapeeth, Akola, MS 444104 India; 2 Entomology Section, College of Agriculture Nagpur, Panjabrao Deshmukh Krishi Vidyapeeth, Akola, MS 440012 India; 3 Agriculture Research Station, Sindewahi, Panjabrao Deshmukh Krishi Vidyapeeth, Akola, MS 441222 India; 4 Department of Agricultural Biotechnology, Dr. Panjabrao Deshmukh Krishi Vidyapeeth, Akola, MS 444104 India; 5 School of Agriculture and Food Sustainability, The University of Queensland, Brisbane, 4067 Australia; 6 Civil Engineering Department, Faculty of Engineering, Minia University, Minia, 61111 Egypt; 7 Structural Diagnostics and Analysis Research Group, Faculty of Engineering and Information Technology, University of Pécs, Pécs, 7622 Hungary; 8 Department of Agricultural Engineering, College of Food and Agricultural Sciences, King Saud University, Riyadh 11451, P.O. Box 2460, Saudi Arabia

**Keywords:** Rice, Insecticide modules, Botanicals, Gall midge, Yellow stem borer, Leaf folder, Integrated Pest Management (IPM), Eco-friendly control

## Abstract

**Background:**

Rice cultivation in eastern Vidarbha region of Maharashtra is seriously affected by insect pests such as yellow stem borer, gall midge, and leaf folder. Farmers in this region mainly depend on repeated application of single chemical insecticides, which has led to the development of insecticide resistance and associated environmental concerns. To address this problem, a field experiment entitled “Comparative evaluation of module-based strategies for controlling lepidopterous and dipterous pests in rice (*Oryza sativa* L.) cultivation” was conducted during *kharif* 2020 and 2021 at the Agriculture Research Station, Bhandara, Dr. Panjabrao Deshmukh Krishi Vidyapeeth, Akola, Maharashtra (India). The study was undertaken to evaluate the effectiveness of different pest management modules in reducing pest incidence and improving the rice grain yield.

**Results:**

The results revealed that all the treatment modules significantly reduced the incidence of major rice pests compared to untreated control. Among the tested modules, M4 comprising chlorantraniliprole 0.4% G, cartap hydrochloride 50% SP, and Dinotefuran 20% SG was found to be the most effective in controlling yellow stem borer, gall midge, and leaf folder. Besides the chemical module, module M1 consisting of Neemazal 1% EC applied at 25–30 days after transplanting (DAT), eucalyptus oil at 45–50 DAT, and cartap hydrochloride 50% SP at 60–65 DAT also showed promising performance against the target pests. Rice grain yield was significantly (*P* < 0.05) higher in both M4 and M1. The study further indicated that botanical-based modules supported comparatively better conservation of natural enemies under field conditions.

**Conclusions:**

Among the evaluated pest management modules, M4 demonstrated the most effective suppression of major rice pests and achieved the highest grain yield. However, M1, integrating botanical formulations such as neem and eucalyptus oil with need-based insecticidal applications, emerged as a safer and more eco-friendly alternative. The study highlights that combining botanicals with selective insecticides can substantially reduce chemical dependence while maintaining effective pest control and crop productivity. These findings provide actionable guidance for farmers and policymakers to promote environmentally responsible pest management practices in rice cultivation systems.

**Supplementary Information:**

The online version contains supplementary material available at https://doi.org/10.1186/s12870-026-09601-8.

## Introduction

Rice (*Oryza sativa* L.) is a vital cereal crop and staple food for more than half of the global population [1–4], playing a central role in food security worldwide. However, rice production is constrained by numerous biotic and abiotic stresses that adversely affect grain yield and quality. Among the biotic factors, insect pests represent a major challenge to sustainable cultivation [5]. Throughout the crop growth cycle, rice is attacked by over 100 insect pest species, of which approximately 20 cause significant economic losses [6–9]. The most destructive and widely distributed pests include the yellow stem borer (*Scirpophaga incertulas*), gall midge (*Orseolia oryzae*), brown planthopper (*Nilaparvata lugens*), white-backed planthopper (*Sogatella furcifera*), and leaf folder (*Cnaphalocrocis medinalis*) [10, 11].

Stem borers remain a persistent threat to rice crops throughout the entire growth cycle, causing continuous damage and yield reduction. In contrast, pests such as plant hoppers and gall midge typically trigger localized outbreaks, yet these can result in severe yield losses in specific regions. If left unmanaged, their populations can multiply rapidly and spread across large areas, underscoring the need for timely and effective control measures.

Globally, lepidopteran stem borers are recognized as some of the most destructive insect pests of rice cultivation [12, 7] reported 25 species of stem borers, primarily belonging to the families *Crambidae* and *Pyralidae*, that are economically important in rice ecosystems. Multiple species often coexist within a region, although one or two tend to dominate [13]. Among these, the yellow stem borer (*Scirpophaga incertulas*) is particularly damaging. After hatching, the larvae bore into rice stems, disrupting the translocation of photosynthates and essential nutrients to the upper plant parts [14]. Infestation results in “dead hearts” during the vegetative stage and “white earheads” during the reproductive stage, both of which cause substantial yield losses and serious economic repercussions for farmers [6].

In the Indian context, the yellow stem borer alone accounts for 2–20% crop damage, with each additional percentage of white earheads corresponding to an estimated 1.3% reduction in rice yield [15]. Both larvae and pupae often coexist in the field, with larvae hiding inside plant stems, making them difficult to control through conventional insecticide sprays [14]. Timely application of botanical and chemical insecticides is therefore critical for effective management of stem borers and other rice pests. Precise scheduling not only reduces chemical inputs but also enhances selectivity and efficiency. Monitoring pest incidence and applying insecticides at the economic threshold level (ETL) or economic injury level (EIL) is essential. For stem borers, researchers have recommended an ETL of 5–10% dead hearts (DH) as a practical indicator for initiating control measures [16].

The rice gall midge (*Orseolia oryzae*) is a major insect pest across Asia and Southeast Asia, and in India its impact is compounded by the presence of five distinct biotypes distributed across different regions. Annual yield losses due to gall midge infestations are estimated at 28–35% [17]. The rice gall fly, belonging to the order *Diptera* and family *Cecidomyiidae*, damages seedlings by inducing the formation of silver shoots [18]. Similarly, the rice leaf folder (*Cnaphalocrocis medinalis*), a lepidopteran pest from the family *Pyralidae*, feeds on unfolding leaves, leaving behind characteristic white streaks [19]. Both pests significantly impair photosynthesis, thereby reducing crop productivity and grain quality.

Insecticides remain a practical and widely adopted approach for managing rice pests, and numerous studies have demonstrated their positive effects on grain yield [20–26]. Their effectiveness, rapid action, ease of use, and cost-efficiency make them valuable tools in pest control. However, indiscriminate or excessive application can lead to serious drawbacks, including the development of insecticide resistance in target pests [27], resurgence of pest populations [28], and outbreaks of secondary pests [29, 30]. Moreover, routine insecticide use poses risks to the environment, human health, and non-target beneficial organisms [31].

Traditionally, rice pest management has relied heavily on the routine application of botanicals and chemical insecticides. However, the continuous and indiscriminate use of chemical control measures has led to multiple challenges for both pest management and the environment [32]. Repeated exposure of insect populations to specific insecticides has accelerated the development of resistance, reducing the efficacy of several compounds [33, 34]. In addition, overreliance on chemicals has contributed to environmental degradation, adverse impacts on beneficial organisms, and potential risks to human health [35–37].

The emergence of pest resistance to synthetic insecticides has become a major challenge, driving global interest in organic and biological alternatives to conventional chemicals. Consequently, researchers are increasingly focused on strategies that reduce pesticide dependence [38]. In this context, environmentally friendly pest management approaches have gained recognition as effective means of controlling rice insect pests while minimizing reliance on synthetic inputs [39].

In India, several pest management modules combining insecticides with locally available botanicals have been developed and tested [40–48]. These studies demonstrate that module-based strategies can effectively break resistance, suppress pest populations, and reduce the negative impacts associated with pesticide use. However, such research remains limited in the Vidarbha region of Maharashtra, where rice is the dominant crop during the *kharif* season, characterized by irregular rainfall and high summer temperatures. Smallholder farmers in this region often face economic constraints in accessing costly insecticides, making botanical and biopesticide-based strategies particularly relevant. These approaches are environmentally sustainable, cost-effective, and form a critical component of integrated pest management (IPM) programs [31].

Given the scarcity of experimental data from Vidarbha, the present study was undertaken to evaluate the effectiveness of different pest management modules in controlling yellow stem borer, gall midge, and leaf folder, while enhancing rice grain yield. It was hypothesized that alternative module-based approaches could reduce reliance on chemical pesticides while maintaining effective pest control and supporting sustainable rice production. Accordingly, the specific objective was to evaluate the effectiveness of different pest management modules in controlling yellow stem borer, gall midge, and leaf folder, and in enhancing rice grain yield. The modules tested combined insecticides with botanicals to achieve effective pest suppression while minimizing environmental and health risks, thereby contributing to sustainable and economically viable rice cultivation systems.

## Materials and mehods

### Experimental details

A two-year field experiment was conducted during the *kharif* seasons of 2020 and 2021 at the research farm of the Agriculture Research Station, Bhandara (Dr. Panjabrao Deshmukh Krishi Vidyapeeth, Akola, Maharashtra) to evaluate module-based strategies for managing lepidopterous and dipterous pests in rice. The trial was laid out in a randomized block design (RBD) with five insecticide and botanical spray modules and an untreated control, replicated four times. Each module was tested in individual plot size of 20 m² (Supplementary Fig. 1). The rice variety ‘PKV-HMT’ was transplanted at a spacing of 20 × 15 cm.

In *kharif* 2020, sowing was done on June 29 and transplanting on August 04, while in *kharif* 2021 sowing was on July 01 and transplanting on July 29. Pest management involved three scheduled sprays applied with a knapsack sprayer at 30, 50, and 65 days after transplanting (DAT). In 2020, sprays were applied on September 3, September 23, and October 8, whereas in 2021 they were applied on August 28, September 17, and October 2. This consistent and well-timed spray schedule was designed to coincide with peak pest incidence and optimize crop protection.

The treatment modules included both botanical and chemical formulations: Neemazal 1% EC (Azadirachtin 1% EC), eucalyptus oil, neem oil, chlorantraniliprole 0.4% G (Ferterra^®^), cartap hydrochloride 50% SP, and dinotefuran 10% SC. Modules were applied in three consecutive sprays at 15-day intervals following pest initiation. Details of insecticides and module composition are presented in Tables 1 and 2.

**Table 1:** Details of botanical and insecticidal formulations used in the study

Sr No	Name of botanicals and insecticides	Trade Name	Company/ Source	Group of insecticides	Formulation	Chemical formula
1	Neemazal	Azadirachtin	-	Botanical	1% EC	C_35_H_44_O_16_
2	Eucalyptus oil	-	-	Botanical	-	C_10_H_18_O
3	Neem oil	-	-	Botanical	-	C_35_H_44_O_16_
4	Chlorantraniliprole	Ferterra	E.I. Dupont Co. Gurgaon	Diamides	0.4% G	C_18_H_14_BrCl_2_N_5_O_2_
			Haryana			
5	Cartap hydrochloride	Caldan	Dhanuka Agritech Ltd.	Thiocarbamate	50% SP	C_7_H_16_ClN_3_O_2_S2
6	Dinotefuran	Osheen	Mitsui Chemicals, Inc	Neonicotinoids	20% SG	C_7_H_14_N_4_O_3_

**Table 2:** Details of different treatment modules evaluated during the study

Sr. No	modules	Details of modules	Dose; ml or g/l
1	M1	Neemazal 1% EC 30 DAT	2.0 ml
		Eucalyptus oil 50 DAT	2.0 ml
		Cartap Hydrochloride 50% SP 65 DAT	2.0 g
2	M2	Neemazal 1% EC 30 DAT	2 ml
		Eucalyptus oil- 50 DAT	2 ml
		Neem oil 65 DAT	10 ml
3	M3	Neemazal 1% EC 30 DAT	2 ml
		Neem oil 50 DAT	10 ml
		Dinotefuran 20% SG 65 DAT	0.3 g
4	M4	Chlorantraniliprole 0.4 G 30 DAT	1 g
		Cartap hydrochloride 50 SP 50 DAT	2 g
		Dinotefuran 20% SG 65 DAT	0.3 g
5	Untreated control	Only Water Spray	-

### Methodology for recoding observations

Pest infestation data were recorded after pest initiation across the evaluated modules. Each module involved three sequential insecticidal applications. The incidence of silver shoots (SS) caused by gall midge and dead hearts (DH) caused by yellow stem borer was assessed by randomly selecting 20 hills using stratified random sampling, one day before and 15 days after each application. Total tiller counts were also recorded during these observations. Similarly, the incidence of white earheads at harvest was recorded along with the number of productive tillers.

For leaf folder infestation, 10 random hills were selected per plot, and the number of damaged leaves (LD) were recorded. Observations were made one day before and seven days after each insecticidal application by counting the total number of leaves.

The percentage incidence of gall midge, yellow stem borer, and leaf folder was calculated using the following formulas:

1 $$\%\:\mathrm{s}\mathrm{i}\mathrm{l}\mathrm{v}\mathrm{e}\mathrm{r}\:\mathrm{s}\mathrm{h}\mathrm{o}\mathrm{o}\mathrm{t}=\:\frac{\mathrm{N}\mathrm{o}.\:\mathrm{o}\mathrm{f}\:\mathrm{s}\mathrm{i}\mathrm{l}\mathrm{v}\mathrm{e}\mathrm{r}\:\mathrm{s}\mathrm{h}\mathrm{o}\mathrm{o}\mathrm{t}\:\mathrm{i}\mathrm{n}\:20\:\mathrm{h}\mathrm{i}\mathrm{l}\mathrm{l}\mathrm{s}}{\mathrm{T}\mathrm{o}\mathrm{t}\mathrm{a}\mathrm{l}\:\mathrm{n}\mathrm{o}.\:\mathrm{o}\mathrm{f}\:\mathrm{t}\mathrm{i}\mathrm{l}\mathrm{l}\mathrm{e}\mathrm{r}\:\mathrm{i}\mathrm{n}\:20\:\mathrm{h}\mathrm{i}\mathrm{l}\mathrm{l}\mathrm{s}}\:\times\:100$$

2 $$\%\:\mathrm{d}\mathrm{e}\mathrm{a}\mathrm{d}\:\mathrm{h}\mathrm{e}\mathrm{a}\mathrm{r}\mathrm{t}=\:\frac{\mathrm{N}\mathrm{o}.\:\mathrm{o}\mathrm{f}\:\mathrm{s}\mathrm{i}\mathrm{l}\mathrm{v}\mathrm{e}\mathrm{r}\:\mathrm{s}\mathrm{h}\mathrm{o}\mathrm{o}\mathrm{t}\:\mathrm{i}\mathrm{n}\:20\:\mathrm{h}\mathrm{i}\mathrm{l}\mathrm{l}\mathrm{s}}{\mathrm{T}\mathrm{o}\mathrm{t}\mathrm{a}\mathrm{l}\:\mathrm{n}\mathrm{o}.\:\mathrm{o}\mathrm{f}\:\mathrm{t}\mathrm{i}\mathrm{l}\mathrm{l}\mathrm{e}\mathrm{r}\:\mathrm{i}\mathrm{n}\:20\:\mathrm{h}\mathrm{i}\mathrm{l}\mathrm{l}\mathrm{s}}\:\times\:100$$

3 $$\%\:\mathrm{w}\mathrm{h}\mathrm{i}\mathrm{t}\mathrm{e}\:\mathrm{e}\mathrm{a}\mathrm{r}\mathrm{h}\mathrm{e}\mathrm{a}\mathrm{d}\mathrm{s}=\:\frac{\mathrm{N}\mathrm{o}.\:\mathrm{o}\mathrm{f}\:\mathrm{w}\mathrm{h}\mathrm{i}\mathrm{t}\mathrm{e}\:\mathrm{e}\mathrm{a}\mathrm{r}\mathrm{h}\mathrm{e}\mathrm{a}\mathrm{d}\mathrm{s}\:\mathrm{i}\mathrm{n}\:20\:\mathrm{h}\mathrm{i}\mathrm{l}\mathrm{l}\mathrm{s}\:\:\:\:\:\:\:}{\mathrm{T}\mathrm{o}\mathrm{t}\mathrm{a}\mathrm{l}\:\mathrm{n}\mathrm{o}.\:\mathrm{o}\mathrm{f}\:\mathrm{p}\mathrm{r}\mathrm{o}\mathrm{d}\mathrm{u}\mathrm{c}\mathrm{t}\mathrm{i}\mathrm{v}\mathrm{e}\:\mathrm{t}\mathrm{i}\mathrm{l}\mathrm{l}\mathrm{e}\mathrm{r}\mathrm{s}\mathrm{n}\:20\:\mathrm{h}\mathrm{i}\mathrm{l}\mathrm{l}\mathrm{s}}\:\times\:100$$

4 $$\%\:\mathrm{D}\mathrm{a}\mathrm{m}\mathrm{a}\mathrm{g}\mathrm{e}\:\mathrm{l}\mathrm{e}\mathrm{a}\mathrm{f}\:\mathrm{f}\mathrm{o}\mathrm{l}\mathrm{d}\mathrm{e}\mathrm{r}=\:\frac{\mathrm{N}\mathrm{o}.\:\mathrm{o}\mathrm{f}\:\mathrm{d}\mathrm{a}\mathrm{m}\mathrm{a}\mathrm{g}\mathrm{e}\mathrm{d}\:\mathrm{l}\mathrm{e}\mathrm{a}\mathrm{v}\mathrm{e}\mathrm{s}\:\mathrm{i}\mathrm{n}\:10\:\mathrm{h}\mathrm{i}\mathrm{l}\mathrm{l}\mathrm{s}}{\mathrm{T}\mathrm{o}\mathrm{t}\mathrm{a}\mathrm{l}\:\mathrm{n}\mathrm{o}.\:\mathrm{o}\mathrm{f}\:\mathrm{l}\mathrm{e}\mathrm{a}\mathrm{v}\mathrm{e}\mathrm{s}\:\mathrm{i}\mathrm{n}\:10\:\mathrm{h}\mathrm{i}\mathrm{l}\mathrm{l}\mathrm{s}}\:\times\:100$$

### Timing of spray applications

Spray applications were scheduled at 30, 50, and 65 days after transplanting (DAT), corresponding to critical crop growth stages and the expected incidence of major rice insect pests under the field conditions of eastern Vidarbha. These timings coincided with the peak activity of yellow stem borer, gall midge, and leaf folder, thereby ensuring timely intervention and effective pest suppression.

### Yield and ICBR analysis

In each experimental plot, the numbers of filled and unfilled grains were recorded, and grain yield was measured on a whole-plot basis. Although additional pests such as leaf folder, leaf hopper, and rice bug were present, their population densities were low relative to stem borers; hence, their impact on yield reduction was not considered in this study. Freshly harvested grains contained approximately 20–22% moisture, while dry grains typically had 12–14% moisture. Accordingly, grain yield values were standardized to a 14% moisture level.

The Incremental Cost–Benefit Ratio (ICBR) was calculated by incorporating the total cost of treatment, including the individual costs of botanical and chemical insecticides as well as labor charges for each spray.

### Statistical analysis

The experimental data collected during *kharif* 2020 and 2021 were analyzed using analysis of variance (ANOVA) appropriate for the randomized block design (RBD). Treatment means were compared at the 5% level of significance (*P* = 0.05). Wherever treatment effects were significant, the standard error of mean [SE(m)] and critical difference (CD) values were calculated to facilitate comparison. All statistical analyses were performed using IBM SPSS Statistics software (Version 22.0) [49].

## Results

### Effect of insecticide and botanical spray modules on the incidence of yellow stem borer during vegetative stage of rice

The data presented in Table 3 indicates that all the treated modules showed statistically significant (*P* < 0.05) superiority over the untreated control module. Cumulative results for all three sprays during the *kharif* 2020 demonstrated significantly (*P* < 0.05) lowest yellow stem borer infestation i.e. dead hearts (DH) in module M4 (7.58% DH), followed by module M1 (9.28% DH) and M3 (9.72% DH). Further, these treated modules, M4, M1, and M3, were statistically at par (*P* ≥ 0.05). In contrast, module M2 showed moderate effectiveness in reducing yellow stem borer infestation (10.10% DH), while significantly (*P* < 0.05) highest infestation (11.91% DH) was observed in untreated control.

**Table 3:** Effect of insecticide and botanical spray modules on the incidence of yellow stem borer on rice during *kharif* 2020 and *kharif* 2021

M. *N*.	Details of Module	Rate g or ml/ha	Stem borer incidence (% Dead hearts)											
			Kharif 2020				Kharif 2021				Pooled Mean			
			Spraying			Cummulative Mean	Spraying			Cummulative Mean	Spraying			Cummulative Pooled
			1st	2nd	3rd		1st	2nd	3rd		1st	2nd	3rd	
			45DAT	65DAT	80DAT		45DAT	65DAT	80DAT		45DAT	65DAT	80DAT	
M1	Botanicals-Insecticides													
	Neemazal 1% EC 25–30 DAT Eucalyptus oil 45–50 DAT Cartap Hydrochloride 50% Sc 60–65 DAT	2.0 2.0 2.0	1.75 (1.50)	10.21 (3.27)	15.86 (4.05)	9.28a (3.13)	5.23 (2.39)	4.55 (2.25)	12.22 (3.57)	7.33b (2.80)	3.25 (1.94)	7.27 (2.74)	14.26 (3.84)	8.26b (2.91)
M2	All Botanicals													
	Neemazal 1% EC 25–30 DAT Eucalyptus oil- 45–50 DAT Neem oil 60–65 DAT	2.0 2.0 10.0	1.63 (1.46)	12.25 (3.57)	16.42 (4.11)	10.10b (3.26)	8.74 (3.04)	6.13 (2.58)	13.79 (3.78)	9.55c (3.17)	4.53 (2.24)	9.15 (3.03)	15.30 (3.98)	9.66b (3.14)
M3	Botanicals-Insecticides													
	Neemazal 1% EC 25–30 DAT Neem oil 45–50 DAT Dinotefuran 20% SG 65–70 DAT	2.0 10.0 0.3	2.02 (1.59)	11.03 (3.40)	16.10 (4.05)	9.72a (3.20)	6.50 (2.65)	5.90 (2.53)	12.33 (3.58)	8.24b (2.96)	3.98 (2.12)	8.38 (2.94)	14.49 (3.87)	8.95b (3.06)
M4	All Insecticides													
	Chlorantraniliprole 0.4 G 25–30 DAT Cartap hydrochloride 50 SP 50–55 DAT Dinotefuran 20% SG 65–70 DAT	1.0 g/m^2^ 2.0 0.3	2.01 (1.58)	6.42 (2.63)	14.29 (3.85)	7.58a (2.84)	3.76 (2.06)	2.49 (1.73)	10.10 (3.26)	5.45a (2.44)	2.77 (1.81)	4.42 (2.20)	12.52 (3.61)	6.57a (2.60)
M5	Untreated control	Water Spray	2.21 (1.68)	13.10 (3.69)	20.36 (4.57)	11.91c (3.54)	12.59 (3.62)	4.55 (3.46)	21.63 (4.70)	15.23d (3.97)	6.95 (2.73)	12.21 (3.56)	20.86 (4.62)	13.32c (3.68)
	‘f’ test		NS	Sig	Sig	Sig	NS	Sig	Sig	Sig	Sig	Sig	Sig	Sig
	SE (*±* M)		-	0.24	0.17	0.13	0.19	0.19	0.15	0.10	0.12	0.16	0.08	0.08
	CD at 5%		-	0.73	0.52	0.40	0.55	0.54	0.46	0.30	0.33	0.46	0.29	0.24
	CV (%)		-	14.37	8.21	8.18	13.05	14.25	7.95	8.42	9.96	10.29	9.79	8.08

*DAT *Days after treatment, *Sig *Significant, *NS *Non-Significant

Figures in parentheses are corresponding values of square root transformation for % Dead heart and % White earhead, n=% Dead heart/% White earhead

Similar results were observed in the *kharif* 2021, where significantly (*P* < 0.05) lowest infestation of yellow stem borer (5.45% DH) was recorded in module M4, followed by module M1 (7.33% DH), M3 (8.24% DH), and M2 (9.55% DH) (Table 2). Module M4 was significantly (*P* < 0.05) superior in reducing yellow stem borer infestation, while the highest infestation (15.23% DH) was observed in module M5 i.e. untreated control.

### Cumulative pooled mean population of yellow stem borer

The analysis of the combined data of yellow stem borer indicates that there was statistically significant (*P* < 0.05) difference among the various treatment modules (Table 3). Module M4 had the significantly (*P* < 0.05) lowest occurrence of yellow stem borer population, with a rate of 6.57% DH. Module M4 performed significantly (*P* < 0.05) better than all the other treatment modules. Module M1 had an incidence of 8.26% DH, while modules M3 and M2 had rates of 8.95% DH and 9.66% DH, respectively. These three treatment modules (M1, M3, and M2) were statistically at par with each other (*P* ≥ 0.05). In contrast, among all the treated modules, module M5 (untreated control) had the highest incidence of yellow stem borer (13.32% DH).

### Effect of insecticide and botanical spray modules on the incidence of yellow stem borer during reproductive stage of rice

During *Kharif* 2020 and 2021, module M4 had significantly (*P* < 0.05) lowest white earhead (WE) infestation, with rates of 8.62% and 10.84% WE, respectively. Following this, module M1 (8.79% and 10.77% WE) and module M3 (10.15% and 11.86% WE) had relatively lower levels of white earhead infestation, and were statistically at par (*P* ≥ 0.05). Module M2 had lower white earhead infestation compared to untreated control, with rates of 12.23% WE (2020) and 15.64% WE (2021). Importantly, module M2 was more effective than untreated control (13.56% and 19.73% WE), which had the highest incidence of white earhead infestation.

### Cumulative pooled mean of white earheads

Data on incidence of white earheads revealed that among all treated modules, module M4 exhibited the significantly (*P* < 0.05) lowest incidence of white earhead infestation, with a rate of 9.72% WE (Table 4). Module M1 and M3 had a slightly higher infestation rate at 9.76% WE and 10.99% WE, respectively. These treated modules (M1 and M3) were statistically at par (*P* ≥ 0.05) with each other. Likewise, treated module M2 had higher infestation of white earheads (13.67% WE) as compare to module M1 and M3. Further, module M2 was significantly (*P* < 0.05) superior over untreated control (16.40% WE) which had highest infestation of white earheads.

**Table 4:** Effect of insecticide and botanical spray modules on the incidence of white earhead of rice during *kharif* 2020 and *kharif* 2021

M.*N*	Details of Module	Rate g or ml/ha	% White earhead		
			Before harvesting		
			2020	2021	Pooled Mean
M1	Botanicals-Insecticides				
	Neemazal 1% EC 25–30 DAT Eucalyptus oil 45–50 DAT Cartap Hydrochloride 50% Sc 60–65 DAT	2.0 2.0 2.0	8.97 (3.08)	10.77 (3.36)	9.76a (3.84)
M2	All Botanicals				
	Neemazal 1% EC 25–30 DAT Eucalyptus oil- 45–50 DAT Neem oil 60–65 DAT	2.0 2.0 10.0	12.23 (3.57)	15.64 (4.02)	13.67b (3.98)
M3	Botanicals-Insecticides				
	Neemazal 1% EC 25–30 DAT Neem oil 45–50 DAT Dinotefuran 20% SG 65–70 DAT	2.0 10.0 0.3	10.15 (3.26)	11.86 (3.52)	10.99a (3.87)
M4	All Insecticides				
	Chlorantraniliprole 0.4 G 25–30 DAT Cartap hydrochloride 50 SP 50–55 DAT Dinotefuran 20% SG 65–70 DAT	1.0 g/m2 2.0 0.3	8.62 (3.02)	10.84 (3.37)	9.72a (3.61)
M5	Untreated control	Water Spray	13.56 (3.72)	19.73 (4.50)	16.40c (4.65)
	‘f’ test		Sig	Sig	Sig
	SE (± M)		0.13	0.10	0.08
	CD at 5%		0.40	0.29	0.29
	CV (%)		7.85	10.02	9.79

*Sig* Significant, *NS* Non-Significant

Figures in parentheses are corresponding values of square root transformation for % Dead heart and % White earhead, n=% Dead heart/% White earhead

### Effect of insecticide and botanical spray modules on the incidence of gall midge of rice during *kharif* 2020 and *kharif* 2021

Different modules had a significant (*P* < 0.05) effect on the incidence of rice gall midge, during both the experimental years (Table 5). All the treated modules were significantly (*P* < 0.05) superior over untreated control module (M5). In *kharif* 2020, significantly (*P* < 0.05) lowest infestation of gall midge was recorded in module M4 (10.93% silver shoot (SS), followed by module M1 (14.54% SS), module M2 (16.73% SS) and M3 (19.21% SS). Of these, module M4 was found to be most effective and significantly superior over all other treated modules. In contrast, the maximal infestation (23.91% SS) of gall midge was observed in untreated control M5. Similarly, in *kharif* 2021, the lowest infestation (17.98% SS) of gall midge was recorded in module M4, followed by module M1 (22.94% SS), module M2 (22.98% SS) and M3 (24.38% SS), while maximal infestation (33.91% SS) of gall midge was observed in untreated control.

**Table 5:** Effect of insecticide and botanical spray modules on the incidence of gall midge on rice during *kharif* 2020 and *kharif* 2021

M.*N*.	Details of Module	Rate g or ml/lit	Gall midge (% Silver Shoot)											
			Kharif 2020				Kharif 2021				Pooled Mean			
			Spraying			Cummulative Mean	Spraying			Cummulative Mean	Spraying			Cummulative Pooled
			1st	2nd	3rd		1st	2nd	3rd		1st	2nd	3rd	
			45DAT	65DAT	80DAT		45DAT	65DAT	80DAT		45DAT	65DAT	80DAT	
M1	Botanicals-Insecticides													
	Neemazal 1% EC 25–30 DAT Eucalyptus oil 45–50 DAT Cartap Hydrochloride 50% Sc 60–65 DAT	2.0 2.0 2.0	17.95 (25.07)	17.22 (24.52)	8.43 (16.88)	14.54b (22.41)	25.20 (30.13)	30.72 (33.66)	13.03 (21.16)	22.94b (28.62)	21.07 (27.32)	24.78 (29.86)	10.44 (18.85)	18.77b (25.67)
M2	All Botanicals													
	Neemazal 1% EC 25–30 DAT Eucalyptus oil- 45–50 DAT Neem oil 60–65 DAT	2.0 2.0 10.0	19.75 (26.38)	18.69 (25.62)	11.74 (20.04)	16.73c (24.14)	27.29 (31.49)	32.40 (34.69)	9.13 (17.59)	22.98b (28.65)	23.45 (28.96)	25.86 (30.57)	10.70 (19.10)	20.00b (26.57)
M3	Botanicals-Insecticides													
	Neemazal 1% EC 25–30 DAT Neem oil 45–50 DAT Dinotefuran 20% SG 65–70 DAT	2.0 10.0 0.3	24.13 (29.42)	20.77 (27.11)	12.74 (20.92)	19.21d (26.00)	28.06 (31.99)	34.93 (36.23)	10.14 (18.57)	24.38c (29.59)	25.93 (30.61)	28.60 (32.33)	11.66 (19.97)	22.06c (28.01)
M4	All Insecticides													
	Chlorantraniliprole 0.4 G 25–30 DAT Cartap hydrochloride 50 SP 50–55 DAT Dinotefuran 20% SG 65–70 DAT	1.0 g/m^2^ 2.0 0.3	15.92 (23.51)	9.76 (18.20)	7.11 (15.46)	10.93a (19.30)	19.75 (26.39)	26.49 (30.98)	7.70 (16.11)	17.98a (25.09)	17.46 (24.70)	19.03 (25.86)	7.33 (15.70)	14.61a (22.47)
M5	Untreated control	Water Spray	26.65 (31.08)	24.11 (29.41)	20.98 (27.26)	23.91 (29.28)	38.31 (38.24)	40.70 (39.64)	22.73 (28.47)	33.91d (35.62)	31.80 (34.32)	33.59 (35.42)	21.71 (27.77)	29.03d (32.60)
	‘f’ test		Sig	Sig	Sig	Sig	Sig	Sig	Sig	Sig	SIG	Sig	Sig	Sig
	SE (*±* M)		1.23	1.72	1.07	0.97	1.93	1.27	0.87	1.02	1.12	1.01	0.77	0.67
	CD at 5%		3.78	5.31	3.30	0.31	5.95	3.91	2.67	3.11	3.45	3.12	2.38	1.99
	CV (%)		9.08	13.86	10.70	7.07	12.23	7.24	8.52	8.25	7.68	9.58	11.64	7.78

*DAT* Days after treatment, *Sig* Significant, *NS* Non Significant

Figures in parentheses are corresponding values of Arc sine transformation, n= % Silver shoot infestation

### Cumulative pooled mean population of gall midge

The pooled data also showed similar results, with module M4 having the significantly (*P* < 0.05) lowest gall midge incidence (14.61% SS), followed by module M1 (18.77% SS) and M2 (20.00% SS) (Table 5). These treated modules did not differ significantly (*P* ≥ 0.05) from each other but were significantly superior than M3 and untreated control. Module M3 was moderately effective (22.06% SS) in managing gall midge incidence, while module M5 i.e. untreated control had the highest gall midge incidence (29.03% SS) among all treated modules.

### Effect of insecticide and botanical spray modules on the incidence of leaf folder of rice during *kharif* 2020 and *kharif* 2021

The incidence of leaf folder population was also significantly (*P* < 0.05) affected by different modules (Table 6). All treated modules were significantly (*P* < 0.05) superior over untreated control module (M5). During *kharif* 2020 and 2021 the least infestation of leaf folder was recorded in module M4 (1.68% and 1.86% LD), followed by module M1 (2.22% and 2.90% LD), module M2 (2.39% and 3.63% LD) and M3 (2.43% and 3.84% LD), respectively. All treated modules were found to be statistically at par (*P* ≥ 0.05) with each other. The maximal infestation of leaf folder was observed in untreated control i.e. 4.02% and 6.62% LD (Table 6), during *kharif* 2020 and 2021, respectively.

**Table 6:** Effect of insecticide and botanical spray modules on the incidence of leaf folder of rice during *kharif* 2020 and *kharif* 2021

M.*N*.	Details of Module	Rate g or ml/ ha	Leaf Folder incidence (% Leaf Damage)											
			Kharif 2020				Kharif 2021				Pooled Mean			
			Spraying			Cummulative Mean	Spraying			Cummulative Mean	Spraying			Cummulative Pooled
			1st	2nd	3rd		1st	2nd	3rd		1st	2nd	3rd	
			37DAT	57DAT	72DAT		37DAT	57DAT	72DAT		37DAT	57DAT	72DAT	
M1	Botanicals-Insecticides													
	Neemazal 1% EC 25–30 DAT Eucalyptus oil 45–50 DAT Cartap Hydrochloride 50% Sc 60–65 DAT	2.0 2.0 2.0	0.80 (1.14)	2.19 (1.64)	2.25 (1.66)	2.22a (1.65)	4.08 (2.14)	2.25 (1.66)	3.55 (2.01)	2.90b (1.84)	2.54 (1.74)	2.23 (1.65)	2.93 (1.85)	2.58b (1.76)
M2	All Botanicals													
	Neemazal 1% EC 25–30 DAT Eucalyptus oil- 45–50 DAT Neem oil 60–65 DAT	2.0 2.0 10.0	0.69 (1.09)	2.29 (1.67)	2.49 (1.73)	2.39a (1.70)	4.54 (2.24)	2.79 (1.81)	4.48 (2.33)	3.63c (2.03)	2.81 (1.82)	2.58 (1.75)	3.50b (2.00)	3.04b (1.88)
M3	Botanicals-Insecticides													
	Neemazal 1% EC 25–30 DAT Neem oil 45–50 DAT Dinotefuran 20% SG 65–70 DAT	2.0 10.0 0.3	0.82 (1.15)	2.47 (1.72)	2.39 (1.70)	2.43a (1.71)	4.20 (2.17)	2.79 (1.81)	4.89 (2.32)	3.84c (2.08)	2.62 (1.77)	2.62 (1.77)	3.70 (2.05)	3.16c (1.91)
M4	All Insecticides													
	Chlorantraniliprole 0.4 G 25–30 DAT Cartap hydrochloride 50 SP 50–55 DAT Dinotefuran 20% SG 65–70 DAT	1.0 g/m^2^ 2.0 0.3	0.80 (1.14)	1.42 (1.39)	1.95 (1.57)	1.68a (1.48)	2.23 (1.65)	2.06 (1.60)	1.67 (1.47)	1.86a (1.54)	1.48 (1.41)	1.79 (1.51)	1.80 (1.52)	1.80a (1.52)
M5	Untreated control	Water Spray	1.89 (1.55)	3.99 (2.12)	4.04 (2.13)	4.02b (2.13)	6.30 (2.60)	6.12 (2.57)	7.13 (2.76)	6.62d (2.66)	4.16c (2.17)	5.17 (2.38)	5.63 (2.48)	5.40d (2.43)
	‘f’ test		Sig	Sig	Sig	Sig	Sig	Sig	Sig	Sig	Sig	Sig	Sig	Sig
	SE (*±* M)		0.08	0.11	0.17	0.09	0.14	0.07	0.11	0.06	0.09	0.07	0.06	0.05
	CD at 5%		0.20	0.28	0.46	0.25	0.42	0.23	0.30	0.18	0.28	0.21	0.17	0.14
	CV (%)		10.96	11.03	17.70	9.69	13.26	8.07	9.03	15.89	10.54	9.50	9.79	8.95

*Sig *Significant, *NS* Non-Significant

Figures in parentheses are corresponding values of square root transformation, n=% Leaf folder damaged leaves

#### Cumulative pooled mean population of leaf folder

Treated module M4 (1.80% LD) was significantly (*P* < 0.05) superior over all treated modules (Table 6). It was followed by module M1 (2.58% LD) and M2 (3.04% LD) which had least number of leaf folder population and these treated modules were significantly at par (*P* ≥ 0.05) with each other. However, module M3 had slightly highest leaf folder incidence (3.16% LD), whereas uncontrol had maximum number of leaf folder infestation (5.40% LD).

### Effect of insecticide and botanical spray modules on the incidence of Coccinellid beetle of rice during *kharif* 2020 and 2021

The population of coccinellid beetles was significantly (*P* < 0.05) influenced by different treatment modules during both years of investigation (Table 7). During *kharif* 2020, the highest cumulative mean population of coccinellid beetles was recorded in the untreated control M5 (0.40 beetles/hill). Among the treated modules, M2 maintained comparatively higher population (0.28 beetles/hill), followed by M3 (0.25 beetles/hill) and M1 (0.24 beetles/hill). The lowest population was observed in module M4 (0.23 beetles/hill), indicating comparatively greater adverse effect on coccinellid beetles.

**Table 7:** Effect of insecticide and botanical spray modules on abundance of Coccinellid beetle of rice during *kharif* 2020 and *kharif* 2021

M.*N*.	Details of Module	Rate g or ml/ ha	Coccinellids/Hills											
			Kharif 2020				Kharif 2021				Pooled Mean			
			Spraying			Cummulative Mean	Spraying			Cummulative Mean	Spraying			Cummulative Pooled
			1st	2nd	3rd		1st	2nd	3rd		1st	2nd	3rd	
			40DAT	60DAT	75DAT		40DAT	60DAT	75DAT		40DAT	60DAT	75DAT	
M1	Botanicals-Insecticides													
	Neemazal 1% EC 25–30 DAT Eucalyptus oil 45–50 DAT Cartap Hydrochloride 50% SP 60–65 DAT	2.0 2.0 2.0	0.08 (0.76)	0.38 (0.94)	0.28 (0.51)	0.24 (0.48)	0.20 (0.77)	0.18 (0.41)	0.23 (0.85)	0.20 (0.84)	0.14 (0.80)	0.28 (0.88)	0.25 (0.87)	0.22 (0.85)
M2	All Botanicals													
	Neemazal 1% EC 25–30 DAT Eucalyptus oil- 45–50 DAT Neem oil 60–65 DAT	2.0 2.0 10.0	0.10 (0.77)	0.43 (0.96)	0.30 (0.55)	0.28 (0.52)	0.25 (0.95)	0.28 (0.52)	0.25 (0.87)	0.26 (0.87)	0.18 (0.82)	0.35 (0.92)	0.28 (0.88)	0.27 (0.88)
M3	Botanicals-Insecticides													
	Neemazal 1% EC 25–30 DAT Neem oil 45–50 DAT Dinotefuran 20% SG 65–70 DAT	2.0 10.0 0.3	0.13 (0.79)	0.38 (0.94)	0.25 (0.50)	0.25 (0.50)	0.15 (0.84)	0.15 90.38)	0.20 (0.84)	0.17 (0.82)	0.14 (0.80)	0.26 (0.87)	0.23 (0.85)	0.21 (0.84)
M4	All Insecticides													
	Chlorantraniliprole 0.4% G 25–30 DAT Cartap hydrochloride 50% SP 50–55 DAT Dinotefuran 20% SG 65–70 DAT	1.0 g/m^2^ 2.0 0.3	0.13 (0.79)	0.33 (0.91)	0.25 (0.50)	0.23 (0.48)	0.15 (0.84)	0.13 (0.35)	0.20 (0.84)	0.16 (0.81)	0.14 (0.80)	0.23 (0.85)	0.23 (0.85)	0.20 (0.83)
M5	Untreated control	Water Spray	0.13 (0.79)	0.60 (1.05)	0.48 (0.68)	0.40 (0.63)	0.48 (0.84)	0.30 (0.54)	0.28 (0.88)	0.35 (0.92)	0.30 (0.89)	0.45 (0.97)	0.38 (0.94)	0.38 (0.94)
	‘f’ test		NS	NS	Sig	Sig	Sig	Sig	NS	Sig	Sig	NS	Sig	Sig
	SE (*±* M)		0.02	0.07	0.05	0.03	0.03	0.05	0.05	0.04	0.03	0.04	0.03	0.01
	CD at 5%		-	-	0.15	0.11	0.08	0.14	-	0.11	0.08	-	0.09	0.05
	CV (%)		14.77	19.04	7.47	13.28	11.60	21.11	12.79	15.58	14.00	13.79	11.94	9.00

*NS* Non-Significant

Figures in parentheses are corresponding values of square root (n+ 0.5) transformation, n= Coccinellids (no./hill)

A similar trend was noticed during *kharif* 2021. The untreated control M5 again registered the highest cumulative mean population (0.35 beetles/hill). Among the treated modules, M2 recorded relatively higher population (0.26 beetles/hill), followed by M1 (0.20 beetles/hill) and M3 (0.17 beetles/hill), whereas module M4 recorded the minimum population (0.16 beetles/hill).

#### Cumulative pooled mean population of Coccinellid beetle of rice

The pooled analysis of both years also revealed significant (*P* < 0.05) variation among the modules (Table 7). The highest cumulative pooled mean population of coccinellid beetles was observed in the untreated control M5 (0.38 beetles/hill). Among the treated modules, M2 recorded comparatively higher population (0.27 beetles/hill), followed by M1 (0.22 beetles/hill) and M3 (0.21 beetles/hill). The minimum pooled population was recorded in module M4 (0.20 beetles/hill), suggesting that insecticide-intensive treatments had comparatively greater impact on coccinellid beetle population.

### Effect of insecticide and botanical spray modules on the incidence of rice spiders during *kharif* 2020 and 2021

The abundance of spiders was significantly (*P* < 0.05) influenced by different treatment modules during both years of experimentation (Table 8). During *kharif* 2020, the highest cumulative mean spider population was recorded in the untreated control M5 (0.63 spiders/hill). Among the treated modules, M2 maintained comparatively higher spider population (0.42 spiders/hill), followed by M1 (0.39 spiders/hill) and M3 (0.37 spiders/hill). The lowest spider population was observed in module M4 (0.29 spiders/hill), indicating comparatively greater reduction in spider abundance under insecticide-intensive treatment.

**Table 8:** Effect of insecticide and botanical spray modules on abundance of Spider of rice during *kharif* 2020 and *kharif* 2021

M.*N*.	Details of Module	Rate g or ml/ ha	Spiders/Hills											
			Kharif 2020				Kharif 2021				Pooled Mean			
			Spraying			Cummulative Mean	Spraying			Cummulative Mean	Spraying			Cummulative Pooled
			1st	2nd	3rd		1st	2nd	3rd		1st	2nd	3rd	
			40DAT	60DAT	75DAT		40DAT	60DAT	75DAT		40DAT	60DAT	75DAT	
M1	Botanicals-Insecticides													
	Neemazal 1% EC 25–30 DAT Eucalyptus oil 45–50 DAT Cartap Hydrochloride 50% Sc 60–65 DAT	2.0 2.0 2.0	0.18 (0.82)	0.50 (1.00)	0.50 (0.78)	0.39 (0.62)	0.25 (0.48)	0.35 (0.92)	0.30 (0.53)	0.30 (0.89)	0.21 (0.84)	0.43 (0.96)	0.40 (0.95)	0.35 (0.92)
M2	All Botanicals													
	Neemazal 1% EC 25–30 DAT Eucalyptus oil- 45–50 DAT Neem oil 60–65 DAT	2.0 2.0 10.0	0.23 (0.85)	0.53 (1.01)	0.50 (0.78)	0.42 (0.64)	0.25 (0.49)	0.40 (0.95)	0.35 (0.58)	0.33 (0.91)	0.24 (0.86)	0.46 (0.98)	0.43 (0.96)	0.38 (0.94)
M3	Botanicals-Insecticides													
	Neemazal 1% EC 25–30 DAT Neem oil 45–50 DAT Dinotefuran 20% SG 65–70 DAT	2.0 10.0 0.3	0.20 (0.84)	0.50 (1.00)	0.40 (0.69)	0.37 (0.61)	0.25 (0.49)	0.33 (0.91)	0.25 (0.49)	0.28 (0.89)	0.23 (0.85)	0.41 (0.96)	0.34 (0.92)	0.33 (0.91)
M4	All Insecticides													
	Chlorantraniliprole 0.4 G 25–30 DAT Cartap hydrochloride 50 SP 50–55 DAT Dinotefuran 20% SG 65–70 DAT	1.0 g/m^2^ 2.0 0.3	0.18 (0.82)	0.45 (0.97)	0.25 (0.57)	0.29 (0.54)	0.33 (0.57)	0.28 (0.88)	0.28 (0.51)	0.28 (0.88)	0.25 (0.87)	0.36 (0.93)	0.24 (0.86)	0.28 (0.89)
M5	Untreated control	Water Spray	0.35 (0.92)	0.78 (1.13)	0.75 (0.93)	0.63 (0.79)	0.33 (0.56)	0.40 (0.95)	0.40 (0.63)	0.38 (0.94)	0.34 (0.92)	0.59 (1.04)	0.58 (1.04)	0.50 (1.00)
	‘f’ test		NS	Sig	Sig	Sig	NS	NS	NS	NS	NS	NS	Sig	Sig
	SE (*±* M)		0.03	0.05	0.05	0.03	0.05	0.03	0.04	0.02	0.03	0.05	0.02	0.02
	CD at 5%		-	0.17	0.14	0.09	-	-	-	-	-	-	-	0.05
	CV (%)		14.85	14.84	13.55	8.92	16.89	19.78	12.67	17.70	15.88	20.18	17.16	9.21

*NS* Non-Significant

Figures in parentheses are corresponding values of square root (n+ 0.5) transformation, n= Spiders (no./hill)

During *kharif* 2021 the untreated control M5 again recorded the highest cumulative mean population (0.38 spiders/hill). Among the treated modules, module M2 registered relatively higher spider population (0.33 spiders/hill), followed by M1 (0.30 spiders/hill) and M3 (0.28 spiders/hill), whereas module M4 recorded the lowest population (0.25 spiders/hill).

#### Cumulative pooled mean population of Spiders of rice

The pooled analysis also shows a parallel pattern among the different treatment modules (Table 8). The highest cumulative pooled mean population of spiders was recorded in the untreated control M5 (0.50 spiders/hill). Among the treated modules, M2 maintained comparatively higher spider population (0.38 spiders/hill), followed by M1 (0.35 spiders/hill) and M3 (0.33 spiders/hill). The lowest pooled spider population was observed in module M4 (0.28 spiders/hill).

### Rice grain yield

**Fig. 1:**
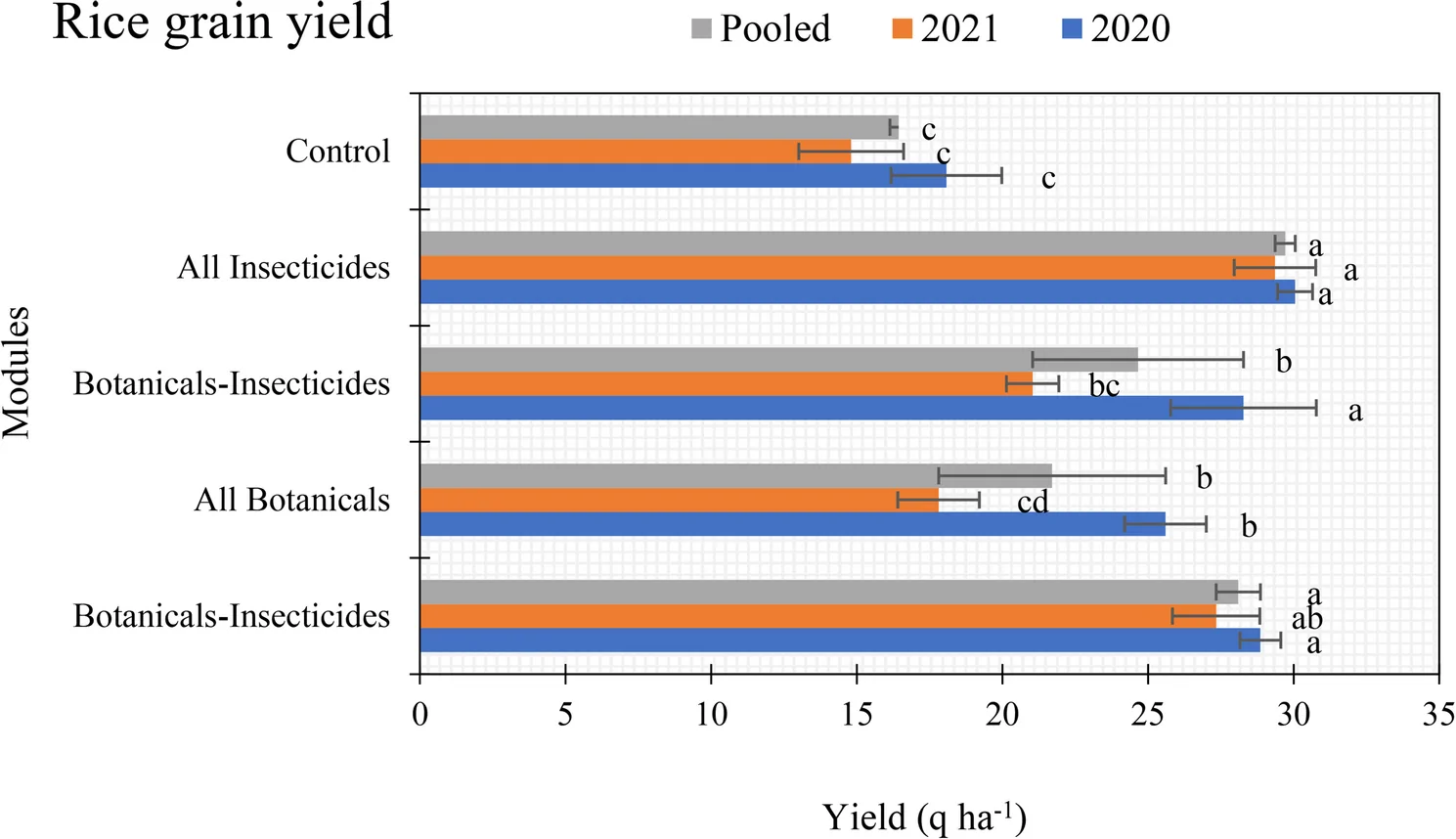
Effect of insecticide and botanical spray modules on yield of rice. The different letters on each bar highlight the significant differences between the modules as per DMRT (*p* < 0.05)

Different treatment modules significantly (*P* < 0.05) affected the rice grain yields during both years (Fig. 1). All treated modules were significantly superior to untreated module M5. In the *kharif* season of 2020 and 2021, treated module M4 was the most effective, with the significantly (*P* < 0.05) highest grain yield (30.05 and 29.36 q ha^− 1^), followed closely by treated module M1 (28.86 and 27.34 q ha^− 1^) and M3 (28.28 and 21.04 q ha^− 1^). During both the years, significantly (*P* < 0.05) lowest grain yield was recorded in untreated control (18.08, and 14.81 q ha^− 1^, respectively).

Pooled data also showed similar result and trend in different modules (Fig. 1). Treated module M4 had the highest grain yield (29.71 q ha^− 1^), followed by treated module M1, with observed grain yield of 28.10 q ha^− 1^. It was observed that, the grain yield of rice in module M4 and M1 was statistically at par (*P* ≥ 0.05). Treated modules M3 and M2 registered moderately high grain yields, at 24.66 q ha^− 1^ and 21.71 qha^− 1^, respectively. All treated modules had significantly highest grain yield of rice compared to untreated control (16.44 q ha^− 1^).

#### Economics and incremental cost-benefit ratio of various modules

The data regarding the Incremental Cost-Benefit Ratio (ICBR) of various modules in rice during the *kharif* seasons of 2020 and 2021 is presented in Table 9 and cost of insecticides used in different modules is given in the Table 10. All modules yielded higher net monetary returns compared to the untreated module. The data in Table 9, concerning increased grain yield over control, showed the highest cost-benefit ratio in the treated module M4, i.e., 1:4.24, which included all insecticides. This was followed by treated module M1 (1:4.05), which included botanicals and insecticides, and then treated module M3 (1:3.28). Module M2 had a moderately high cost-benefit ratio. The lowest cost-benefit ratio was recorded in untreated control.

**Table 9:** Effect of different modules on incremental cost benefit ratio (ICBR)

M.*N*	Treatments	Total cost for 3 spraying (Rs)	Pooled Yield (q/ha)	Yield increased over control (q/ha)	Value of increased yield (Rs)	Incremental Benefit (Rs)	ICBR	Rank
M1	Botanicals-Insecticides	7180	28.10	11.66	29,150	21,970	4.05	2
M2	All Botanicals	6330	21.71	5.27	13,175	6845	2.08	4
M3	Botanicals-Insecticides	6255	24.66	8.22	20,550	14,295	3.28	3
M4	All Insecticides	7670	29.71	13.01	32,525	24,855	4.24	1
M5	Control	-	16.44	0	0	0	0	5

**Table 10:** Effect of different modules on incremental cost benefit ratio (ICBR)

M.*N*		Modules																	Total cost for 3 spraying A + B+C
		Application of Insecticides																	
	First spray	Qty. per ha	Rate per kg or lit.	Cost of modules			Second spray	Qty. per ha	Rate per kg or lit.	Cost of modules									
				Cost of insecticides (Rs /ha)	Labour cost and Spray pump charges (Rs/ha)	Total Cost (Rs/ ha) A				Cost of insecticides (Rs/ha)	Labour cost and Spray pump charges (Rs/ha)	Total Cost (Rs/ ha) B	Third spray	Qty. per ha	Rate per kg or lit.	Cost of insecticides (Rs/ha)	Labour cost and Spray pump charges (Rs/ha)	Total Cost (Rs/ ha) C	
M1	Neemazal 1% EC	1 lit./ha	Rs. 600 /Litre	600	1260	1860	Eucalyptus oil	1 lit. /ha	Rs. 1200 /Litre	1200	1260	2460	Cartap hydrochloride 50% SP	1 kg /ha	Rs. 1600 /kg	1600	1260	2860	7180
M2	Neemazal 1% EC	1 lit./ha	Rs. 600 /Litre	600	1260	1860	Eucalyptus oil	1 lit. /ha	Rs. 1200 /Litre	1200	1260	2460	Neem oil	5 lit./ha	Rs. 150 /Litre	750	1260	2010	6330
M3	Neemazal 1% EC	1 lit./ha	Rs. 600 /Litre	600	1260	1860	Neem oil	5 lit. /ha	Rs. 150 /Litre	750	1260	2010	Dinotefuran 20% SG	150 g/ha	Rs. 750 /100 g	1125	1260	2385	6255
M4	Chlorantraniliprole 0.4G	10 kg /ha	750/4 kg	1875	550	2425	Cartap hydrochloride 50% SP	1 kg /ha	1600/ kg	1600	1260	2860	Dinotefuran 20% SG	150 g/ha	Rs. 750 /100 g	1125	1260	2385	7670
M5	Control	--		--	--	--	Control	--	--	--	--	--	Control	--	--	--	--	--	--
Cost of Insecticides																			
SN	Insecticides required/ha			Cost (Rs)			* Standard Spray Volume – 500 lit./ha. * Labour charges for spray- 4 labour/ha @ Rs. 275day * 4 Knapsack Sprayer Pump Rent -@Rs. 40/day ✥ Market price of rice grain @Rs. 2500/q												
1	Neemazal 1% EC			Rs. 600/Litre															
2	Eucalyptus oil			Rs. 1200/Litre															
3	Cartap hydrochloride 50% SP			Rs. 1600/Kg															
4	Neem oil			Rs. 150/Litre															
5	Dinotefuran 10% SC			Rs. 750/100 g															
6	Chlorantraniliprole 0.4G			Rs. 750/4 kg															

## Discussion

Fertilizer and irrigation management, as well as the use of agrochemicals in rice cultivation, have all contributed to increased production. However, they have further intensified the problem of insect pests [50]. One of the primary challenges faced in rice cultivation is the over-dependence on single chemical insecticide that led to several concerns, chiefly the development of resistance among insect populations [27, 33, 34]. This phenomenon occurs due to repeated exposure to specific insecticides, rendering them less effective over time.

The results obtained in our study highlight the significance of Module M4, which employed the insecticides Chlorantraniliprole 0.4% G, Cartap hydrochloride 50% SP, and Dinotefuran 20% SG. This module was notably more effective in managing yellow stem borer, gall midge, and leaf folder populations and increasing the yield of rice compared to other treated modules. The sequential application of three insecticides i.e. Chlorantraniliprole, Cartap hydrochloride, and Dinotefuran the components of the insecticide spray module with different modes of action, played a pivotal role in their effectiveness. Chlorantraniliprole, for instance, acts as a ryanodine receptor modulator, disrupting the normal muscle function in insects specially in lepidopterous pests and ultimately leading to paralysis and death [51–53]. Cartap hydrochloride functions as a nerve toxin, inhibiting the transmission of nerve impulses in pests [54]. Dinotefuran, on the other hand, is a neonicotinoid insecticide that targets the insect’s nervous system by binding to nicotinic acetylcholine receptors, leading to paralysis and death [55, 56]. The combined action of these three components in M4 creates a multifaceted attack on the pests, minimizing the risk of resistance development and ensuring a highly efficient approach to pest management. With the sets of these three insecticides apart their distinct modes of action and sequential application reduce the likelihood of resistance development in pest populations. These outcomes align with previous research studies [26, 57–60], indicating that insecticides effectively mitigate the damage caused by rice stem borers, ultimately enhancing crop yields. A study by [61] supports the effectiveness of the insecticide dinotefuran at a rate of 150 g ha^–1^ against rice stem borers, corroborating the results of the present study. Further, studies conducted by [62, 63] and [11] explored the efficacy of various compounds and insecticides in controlling rice gall midges. These studies consistently found that certain compounds, such as Cartap hydrochloride 50 SP and Chlorantraniliprole 4% GR, were effective in reducing gall midge infestations.

Further, Module M4 exhibited the highest efficacy in reducing pest infestations and achieving the highest grain yields (29.71 q ha^− 1^), although it had a comparatively higher cost-benefit ratio (1:4.24). This difference could be attributed to potential discrepancies in critical dose computation or variations in market prices for new insecticides. This notion supports the research of [64], who reported that the new 0.4% GR formulation of chlorantraniliprole at 40 g hm^–2^, (marketed as Fertara) effectively manages rice stem borers and yields the highest cost-benefit ratio. Similarly [65], reported the efficacy of Chlorantraniliprole in reducing the percentage of dead hearts and white ear heads in rice. Additionally [66], found that chemical modules containing Cartap hydrochloride were more effective in minimizing damage caused by the yellow stem borer. Few more studies like [67, 68] and [69], found that certain insecticides, such as Cartap hydrochloride 50 SP, were effective in managing leaf folders, supporting our results. These findings are consistent with studies conducted by [11, 64–66, 70] and [71], which all reported favorable results in terms of pest management, yield improvement, and cost-benefit ratios when specific insecticides and integrated pest management approaches were employed.

However, reliance on synthetic pesticides has been associated with serious drawbacks, including deterioration of soil and water quality, contamination of natural water sources, adverse impacts on human health, and disruption of plant and animal life. These detrimental effects threaten agro-biodiversity, biosafety, and overall human well-being [37, 72, 73].

Considering these challenges, our study explored alternative pest management approaches aimed at reducing dependence on chemical insecticides while promoting environmentally sustainable and economically viable methods. By integrating botanicals and biopesticides into module-based strategies, the present study sought to contribute to sustainable rice production systems that balances effective pest control with ecological and human safety. We found that, Modules M1, M3, and M2 also showed moderate efficacy in controlling these pests and were comparable to each other in terms of effectiveness. The spray module M1, which integrated botanicals (Nemazal and Eucalyptus oil) alongside Cartap Hydrochloride 50% SC, exhibited adequate efficiency in pest control with maximum abundance of natural enemies among treated modules. Module M1 substantially decreased DHs ranging from 1.75 to 14.26%, as well as showed a significant reduction in whiteheads (WHs) by 8.97 to 10.77%, compare to control. Moreover, these treatments (M1, M2 and M3) resulted in yield increases of 5.3 to 11. 7%, compared to control. These outcomes are essential for understanding the role of botanicals in pest management. Neemazal (1% EC) derived from the neem tree (*Azadirachta indica*), is a botanical insecticide known for its disruption of the hormonal balance in insects. It affects their feeding, moulting, fertility, and can act as a repellent. However, the mode of action of neem-based products tends to be gradual, requiring time to disrupt the pests’ life cycle and populations. Another botanical used was Eucalyptus Oil recognized for its repellent effects, primarily deterring insects from settling on treated crops. Its mode of action is preventive and might not directly kill existing pests, but it complements the action of Neemazal and Cartap Hydrochloride. The moderate efficiency observed in module M1 suggests that combining botanicals and chemicals can also effectively restrict the growth of insect-pests.

The research literature highlights a concerning trend in pests developing resistance to synthetic insecticides, necessitating the investigation of organic and biological alternatives to these chemicals. As a result, researchers worldwide are increasingly looking for ways to reduce reliance on pesticides [38]. While agrochemicals have undoubtedly increased agricultural production, their indiscriminate application, both intensively and extensively, has resulted in several issues. These include environmental degradation, such as contamination of water, air, and soil, toxicity to non-target organisms, risks to human health from agrochemical residues in food, and decreased chemical efficacy due to pest resistance development [37, 72, 73]. The search for effective and environment friendly pest control methods has intensified, with biological control based on natural enemies and the use of plant-derived natural products emerging as critical strategies. Botanical pesticides are a promising alternative to conventional chemicals in crop protection systems [72–78]. Several approaches can be taken to promote sustainable agriculture. As a result, this study advocates using botanical pesticides in conjunction with insecticides in pest management strategies to sustain crop productivity over time while mitigating environmental degradation. The abundance of plants that serve as sources of botanical pesticides, combined with their multiple uses such as medicinal, culinary, ornamental, and fodder, makes them easily accessible and cost-effective for agricultural systems [79, 80]. Commercial botanical pesticides such as pyrethrum, neem, and sabadilla are among the least toxic, especially to non-target organisms like pollinators and fish, increasing their effectiveness and acceptability in sustainable crop protection [81]. Furthermore, botanical pesticides leave minimal residues on crops and the environment, which helps to conserve the environment and protect consumers [82]. The biochemical interaction between botanical pesticides and pests reduces the possibility of pest resistance development [83, 84]. Furthermore, the target specificity of plant-based chemical compounds in extracts and essential oils protects non-target organisms, including beneficial insects such as pollinators and predators [85, 86]. The success of this integrated approach emphasizes the importance of diversifying pest control strategies to improve efficacy and mitigate the adverse effects of pesticide resistance.

Stem borer infestation adversely affects normal plant growth and development by damaging the vascular tissues and disrupting the translocation of water, nutrients, and photosynthates within the rice plant. The characteristic symptoms such as deadhearts during the vegetative stage and white earheads during the reproductive stage directly reduces productive tillers and grain formation, ultimately resulting in yield reduction [6]. White earhead damage is considered more detrimental because compensation by the plant at the reproductive stage is limited. Earlier studies have also reported that stem borer injury negatively affects plant vigour, photosynthetic activity, and grain filling in rice crops. However, rice plants may exhibit certain compensatory responses such as production of new tillers and redistribution of assimilates under moderate levels of damage. Similar observations on compensatory tillering and plant response to stem borer injury were reported by [87] and [88]. Effective suppression of stem borer infestation in the present study therefore contributed to improved plant health and higher grain yield under treated modules.

The population of natural enemies, particularly spiders and coccinellid beetles, varied among the different pest management modules evaluated during the study. In general, the untreated control plots recorded the highest abundance of natural enemies during both years of experimentation. This may be attributed to the absence of pesticide application, which allowed uninterrupted survival and multiplication of beneficial arthropods in the rice ecosystem. Similar observations have been reported by [46, 89–91] that non-treated rice fields often support comparatively higher populations of predators and parasitoid due to the availability of prey and the absence of chemical disturbance.

Among the treated modules, comparatively higher populations of spiders and coccinellid beetles were observed in the botanical-based modules, especially M2, followed by M1 and M3. The comparatively safer effect of these modules on beneficial organisms may be associated with the relatively lower toxicity and biodegradable nature of botanical products such as neem-based formulations and eucalyptus oil. Botanicals generally exhibit selective action against insect pests and are known to degrade rapidly in the environment, thereby reducing their adverse impact on non-target organisms. These findings are in line with the studies of [46, 89–92].

The practical applicability of different modules used in the present study was considered while recommending pest management strategies. Although module M4 recorded superior pest suppression and grain yield, the comparatively lower input cost and easy availability of botanical products used in module M1 make it more suitable and affordable for small and marginal farmers. Therefore, integration of botanicals with selective insecticides can provide an economically feasible and environmentally safer option for sustainable rice pest management under field conditions.

## Conclusion

The study demonstrated that module-based pest management strategies effectively reduced the incidence of major rice pests namely yellow stem borer, gall midge, and leaf folder while enhancing grain yield under field conditions. Among the tested modules, M4 consistently outperformed others, achieving the highest pest suppression and grain yield. Botanical-based modules, particularly M1, also provided substantial pest reduction with minimal adverse effects on natural enemies, highlighting their eco-friendly potential.

Importantly, modules integrating botanicals with need-based insecticidal applications maintained a better balance between pest control and natural enemy conservation, offering a more sustainable alternative to sole reliance on chemical insecticides. These findings suggest that farmers in rice-growing regions particularly under sub-tropical Indian conditions can adopt M4 as a highly effective option, while botanical-based modules such as M1 serve as cost-effective and environmentally safer choices where reduced chemical use is prioritized.

Overall, the integration of botanicals with selective insecticides emerges as a practical component of Integrated Pest Management (IPM), supporting both productivity and ecological safety. Wider field validation across diverse agro-climatic zones will further strengthen the applicability of these strategies for sustainable rice cultivation.

## Supplementary Information

Supplementary Material 1.

## Data Availability

The datasets used and/or analysed during the current study are available from the corresponding author on reasonable request.

## Abbreviations

ANOVA: Analysis of Variance
CD: Critical Difference
DAT: Days After Transplanting
DH: Dead Hearts
DMRT: Duncan’s Multiple Range Test
EC: Emulsifiable Concentrate
g: Gram
ha: Hectare
ICBR: Incremental Cost Benefit Ratio
IPM: Integrated Pest Management
kg: Kilogram
LD: Leaf Damage
M1–M5: Treatment Modules
MoA: Mode of Action
P: Probability Level
q: Quintal
RBD: Randomized Block Design
Rs.: Indian Rupees
SC: Suspension Concentrate
SE(m): Standard Error of Mean
SG: Soluble Granule
SP: Soluble Powder
SS: Silver Shoots
WE: White Earheads
