# Supplementary material for: Comparative evaluation of module-based strategies for controlling lepidopterous and dipterous pests in rice (Oryza sativa L.) cultivation
Source: BMC Plant Biol. 2026 Aug 6;26:1517. doi: 10.1186/s12870-026-09601-8 (PMC13540895; doi:10.1186/s12870-026-09601-8)
Supplement: Supplementary file 1 [file 12870_2026_9601_MOESM1_ESM.docx]

**Supplementary Fig 1: Layout Plan of Experimental Field**
